# Dynamics of glucose concentration during the initiation of ketogenic diet treatment in children with refractory epilepsy: Results of continuous glucose monitoring

**DOI:** 10.1002/epi4.12778

**Published:** 2023-07-04

**Authors:** Klára Brožová, Juraj Michalec, Marek Brabec, Petra Bořilová, Pavel Kohout, Jan Brož

**Affiliations:** ^1^ Department of Pediatric Neurology Thomayer University Hospital Prague Czech Republic; ^2^ Third Medical Faculty Charles University Prague Czech Republic; ^3^ Department of Internal Medicine, Second Faculty of Medicine Charles University Prague Czech Republic; ^4^ Institute of Computer Science Academy of Science of the Czech Republic Prague Czech Republic; ^5^ Department of Internal Medicine Third Faculty of Medicine Charles University and Thomayer University Hospital Prague Czech Republic; ^6^ Center of Nutrition Thomayer University Hospital Prague Czech Republic

**Keywords:** censored data, continuous glucose monitoring, epilepsy, glucose concentration, ketogenic diet

## Abstract

**Objective:**

The ketogenic diet (KD) is a diet low in carbohydrates and rich in fats which has long been used to treat refractory epilepsy. The metabolic changes related to the KD may increase the risk of hypoglycemia, especially during the first days. The study focused on the impact of KD initiation on glycemia in non‐diabetic patients with refractory epilepsy.

**Methods:**

The subjects were 10 pediatric patients (6 boys, mean age 6.1 ± 2.4 years), treated for intractable epilepsy. Blinded continuous glucose monitoring system (CGM) Dexcom G4 was used. Patients started on their regular diet in the first 36 hours of monitoring, followed by an increase in lipids intake and a gradual reduction of carbohydrates (relations 1:1; 2:1; 3:1; 3.5:1). We analyzed changes in glycemia during fat: nonfat ratio changes using a generalized linear model.

**Results:**

The mean monitored time per person was 6 days, 10 hours and 44 minutes. The mean ± SD glycemia for the regular diet was 4.84 ± 0.20 mmol/L, for the carbohydrates/fat ratio of 1:1 it was 4.03 ± 0.16, for the ratio of 2:1 it was 3.57 ± 0.10, for the ratio 3:1 it was 3.39 ± 0.13 and for the final ratio of 3.5:1 it was 2.79 ± 0.06 mmol/L (*P* < 0.001). The portions of time spent in glycemia ≤3.5 mmol/L (≤2.5 mmol/L respectively) were: on the normal diet 0.88% (0.31%) of the monitored period, during 1:1 KD ratio 1.92% (0.95%), during 2:1 ratio 3.18% (1.02%), and during 3:1 and 3.5:1 ratios 13.64% (2.36%) of the monitored time (*P* < 0.05).

**Significance:**

Continuous glucose monitoring system shows the dynamic of glucose concentration in ketogenic diet treatment initiation. It may be a useful tool to control the effects of this diet on glucose metabolism, especially in hypoglycemia detection.

**Key points**

- Our results show a consistent trend of decreasing glycemic values with increasing fat: nonfat ratio in ketogenic diet
- Risk of hypoglycemia (<3.5 mmoL/L) as well as severe hypoglycemia (<2.5 mmoL/L) increased markedly in higher fat: nonfat ratio
- Blood glucose levels should be monitored carefully in epilepsy patients during the first days of ketogenic diet
- The absence of signs of hypoglycemia might be associated with the syndrome of impaired hypoglycemia awareness which is well‐known in patients with diabetes mellitus

## 1 INTRODUCTION

The ketogenic diet (KD) is an effective treatment for intractable epilepsy not only in children.^1^ It is based on a high proportion of fat, a low proportion of carbohydrates and an amount of protein adequate to the needs of the patient.^2^ Although the mechanisms of its action are not fully understood, it has been shown that KD induces a small reduction in glycolysis, concomitant with an increase in non‐glucose sources of fuel through the oxidation of fatty acids (FA) and ketone bodies. Thus, glycolytic restriction, among other factors, may be an important mechanism mediating the anti‐seizure properties of the KD.^3^

Common side‐effects of KD include nausea and vomiting, acidosis, dehydration, and hypoglycemia.^1^

Although transient hypoglycemia during the introduction of the KD is well documented,^4^, ^5^, ^6^ and longer term hypoglycemic episodes were found in several KD patients^7^, ^8^ suggesting substantial changes in glucose concentrations, detailed dynamics of glucose concentration during KD treatment has never been studied.

Periods of hypoglycemia can be identified by the presence of hypoglycemic symptoms and/or random blood glucose monitoring. Continuous glucose monitoring (CGM) is a method of tracking glucose levels in subcutaneous tissue throughout the day and night. CGM systems take glucose measurements usually at regular intervals of 5 minutes. These devices are among others^9^ used by insulin‐treated diabetes patients^10^ who are not well metabolically controlled, in order to improve their glycemic values; but in general, may be used for glycemic monitoring of any person.^11^

The aim of this study is to describe the glucose concentration changes before the KD initiation and during the first 5 days of its use in children with intractable epilepsy.

To the best of our knowledge, this is the first systematic use of CGM in non‐diabetic children patients with KD.

## 2 MATERIALS AND METHODS

### 2.1 Study design

This single‐center, prospective study was conducted at the Department of Pediatric Neurology, Thomayer Hospital, Prague, Czech Republic between May 16, 2019, and August 31, 2021.

The study was approved by the institutional ethics committee (Thomayer Hospital, No‐19‐15), and informed consent was obtained from the guardians of all the participants. The study was conducted in accordance with the ethical principles of the Declaration of Helsinki.

### 2.2 Participants

We enrolled 10 children aged between 2 years and 18 years (4 females), with drug‐resistant epilepsy (treated with 1–3 antiseizure medications) requiring KD therapy, and without any previous experience with KD, seven with impaired intellectual capacity. Children were excluded if they had absolute contraindications of KD (children with metabolic disorders that impair energy production from lipids^12^ and conditions that could affect glycemic levels, such as diabetes mellitus, impaired glucose tolerance, and hypertriglyceridemia).

### 2.3 Study procedure

#### 2.3.1 Ketogenic diet protocol

The classic KD protocol with a fat: nonfat ratio of 1:1, 2:1, 3:1, and 3.5:1 increased gradually day by day^12^ was used in the study. The patient‐tailored KD for each individual child was designed by clinicians, guardians, and dieticians within 2 weeks before the study initiation. The KD was introduced in out‐patients settings. Participants were also prescribed sugar‐free multivitamins and mineral supplements.

#### 2.3.2 Glycemia and other biochemical measurements

Continuous glucose monitoring system was initiated 36 hours prior to the start of therapy and continued for the following 7 days. The continuous glucose monitoring system (CGM) Dexcom™ G4 Platinum CGM System® (Dexcom, Inc.) which measures glucose concentrations from interstitial fluid in subcutaneous tissue in the range of 2.2–22.0 mmol/L every 5 minutes, was used. The CGM system was “blinded”, thus parents or staff were not able to see glycemic values during the monitoring period. The CGM system must be calibrated by entering 2 glucose readings per day. These were obtained using a portable glucometer (Optium Neo; Abbott Laboratories). Portable glucometers are designed to meet the ISO 15197:2013 standard and must meet these criteria: compared to a traceable laboratory method at least 95% of BGMS results have to be within ±0.83 mmol/L at glucose concentrations <5.5 mmol/L and within ±15% at ≥5.5 mmol/L.^13^

Cholesterol and triglyceride analyses were performed in the hospital biochemical laboratory. CGM data were derived using Dexcom Studio Software, version 12.0.2.2 Blood ketones were measured with FreeStyle Optium Neo ketone meter (Abbott Laboratories), expressed in mmol/L.

### 2.4 Study outcomes

The primary outcomes were the mean morning fasting glycemia and mean glycemia during the whole monitoring related to each particular fat: nonfat ratio. The secondary outcome was the percentage of time spent in hypoglycemic levels: glycemia ≤3.5 mmol/L (≤2.5 mmol/L respectively).

Morning fasting glycemia was defined as glycemia before the first meal in period between 5.00–6.00 o'clock am.

### 2.5 Statistical analysis

We analyzed and summarized the data obtained from the CGM monitor using generalized linear modeling with Gaussian likelihood. Because the CGM has a lower limit for the glycemia values it can report (namely 2.21 mmol/L), instead of reporting very low glyceamia values precisely, it just issues a statement that the glycemia is lower than 2.21 mmol/L instead of giving a precise (point) glycemia value. This is an instance of left censoring in the sense of Survival analysis.^14^, ^15^ Since the glycemia values encountered in this study are generally low, censoring is not so rare and cannot be safely omitted without biasing the analysis. Therefore, we analyzed the glycemic data as (potentially) censored gaussian data via techniques common in Survival analysis (specifically, we used survival package and function survey in r environment).^16^ Further, we summarized the distribution of glycemia via histograms and investigated how the distribution changes under different KD ratios via kernel density estimates.^17^

## 3 RESULTS

### 3.1 Participants' characteristics and treatment details

The subjects were 10 pediatric patients, 6 boys, mean age 6.1 ± 2.4 years. Their characteristics are shown in Table 1.

**TABLE 1:** Participants' characteristics.

Subject	1	2	3	4	5	6	7	8	9	10
Age (years)	6	7	3	9	9	4	2	6	6	9
Weight	25.5	19.5	13.5	23	27	15.3	13.5	23	15.5	21
Height (cm)	116	110	94	116	142	103	89	124	104	128
Sex	M	M	F	F	M	F	M	M	M	F
Energy before the study (kcal/kg)	62	72	74	78	62	60	88	107	72	65
Energy during the study (kcal/kg)	62	72	62	65	62	59	80	97	66	65
Ketones‐maximal value reached (mmol/L)	4.5	4.3	4.8	6.2	5.8	4.6	5.8	6.6	6.3	5.3
Cholesterol value (mmol/L)	6.5	5.6	3.5	4.2	4.7	4.8	3.9	5.7	4.6	5.9
Triglycerides value (mmol/L)	0.77	1.82	0.92	0.9	0.79	1.57	0.9	0.99	2	1.03

### 3.2 Glucose concentrations

**FIGURE 1:**
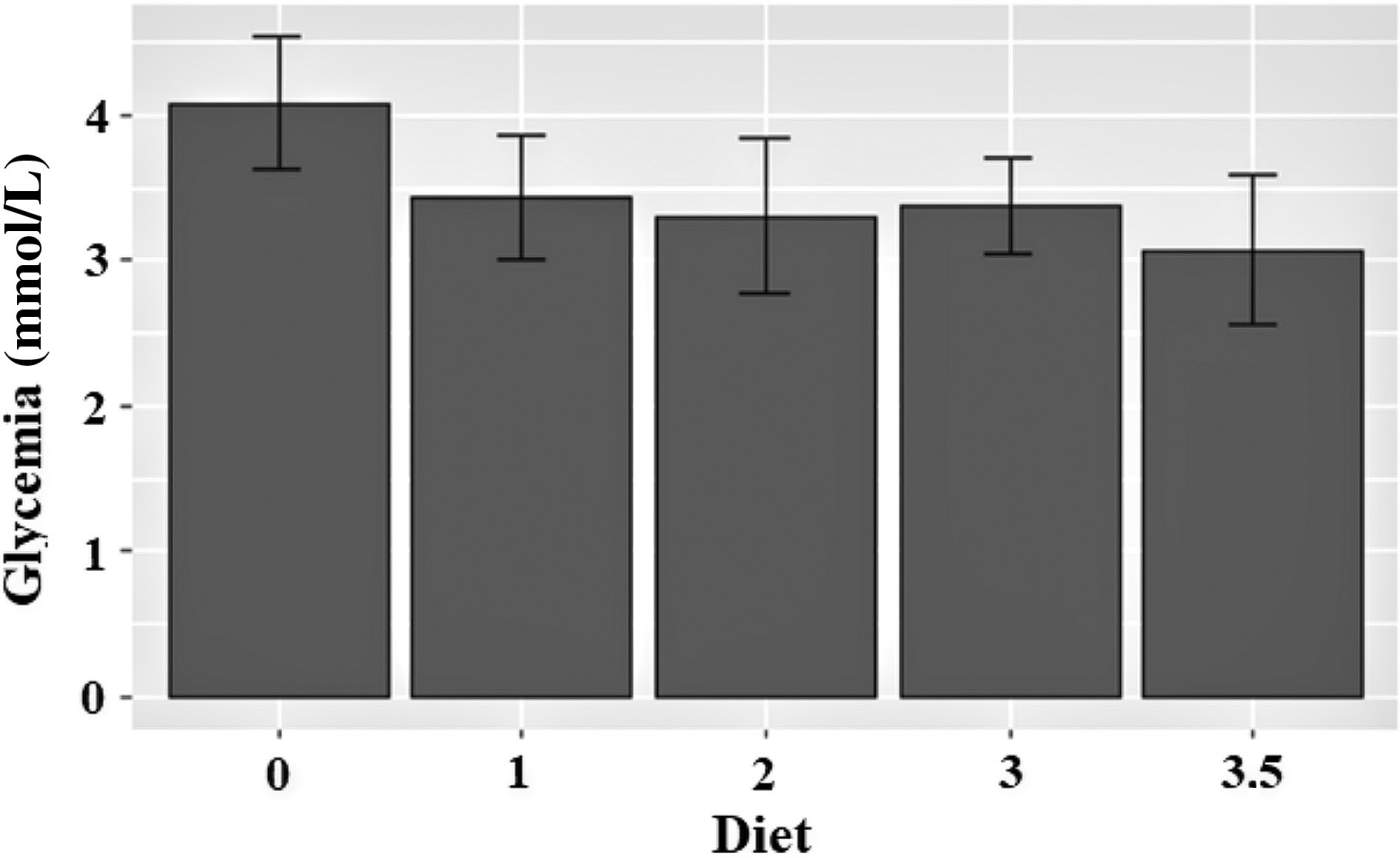
Model‐based estimate of mean fasting glycemia for each ketogenic diet (KD) ratio. The figure shows model estimates of expected fasting glycemia values for different KD ratio groups. These are based on maximum likelihood estimates (MLE's) taking into account (left) censoring (and not raw data which would provide a biased picture due to the censoring). Left censoring is induced by the fact that the sensor has a lower threshold below which it does not report the exact glycemia value but only signals that the glycemia was below the threshold. Diet: 0 = normal; 1 = 1:1; 2 = 1:2; 3 = 1:3; 3.5 = 1:3.5.

The estimated mean monitored time per person was 6 days, 10 hours and 44 minutes. The mean ± SD fasting glycemia for the regular diet was 4.08 ± 0.23 mmol/L, for the fat: nonfat ratio of 1:1 it was 3.4 ± 0.22, for the ratio of 1:2 it was 3.3 ± 0.27, for the ratio 1:3 it was 3.38 ± 0.16 and for the final ratio of 1:3.5 it was 3.1 ± 0.26 mmol/L (*P* < 0.001; Figure 1).

**FIGURE 2:**
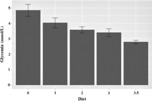
Model based estimate of mean fasting glycemia for each particular ketogenic diet (KD) ratio. The figure shows model estimates of expected all glycemia values for different KD ratio groups. These are based on maximum likelihood estimates (MLE's) taking into account (left) censoring (and not raw data, which would provide a biased picture due to the censoring). Diet: 0 = normal; 1 = 1:1; 2 = 1:2; 3 = 1:3; 3.5 = 1:3.5.

The estimated mean ± SD glycemia for the regular diet was 4.84 ± 0.20 mmol/L, for the carbohydrates/fat ratio of 1:1 it was 4.03 ± 0.16, for the ratio of 1:2 it was 3.57 ± 0.10, for the ratio 1:3 it was 3.39 ± 0.13 and for the final ratio of 1:3.5 it was 2.79 ± 0.06 mmol/L (*P* < 0.001; Figure 2). The portions of time spent in glycemia ≤3.5 mmol/L (≤2.5 mmol/L respectively) were: on the regular diet 0.88% (0.31%) of the monitored period, during 1:1 KD ratio 1.92% (0.95%), during 1:2 ratio 3.18% (1.02%), and during 1:3 and 1:3.5 ratios 13.64% (2.36%) of the monitored time (*P* < 0.05; Figure 2). Participants or caregivers did not report any symptoms of hypoglycemia.

### 3.3 Ketones concentrations

During the KD all the participants reached the required levels of ketones. The highest levels reached are shown in Table 1.

**FIGURE 3:**
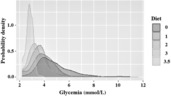
The kernel estimates of glycemia probability density function for each ketogenic diet (KD) ratio. the figure provides a complete look at the probability distribution of glycemia values in different KD groups (not just at mean values as in Figures 1 and 2). The distribution is depicted by standard kernel probability density curve estimate. Inspection of the plot allows for appreciation of the changes in glycemia distribution spread and shape (increasing asymmetry − skewness) with increasing KD ratio. Diet: 0 = normal; 1 = 1:1; 2 = 1:2; 3 = 1:3; 3.5 = 1:3.5.

The kernel density estimate for each particular KD ratio is shown in Figure 3.

## 4 DISCUSSION

Our results showed the significant differences in mean glucose concentrations related to each lipid/sacharide ratio positively correlated with the amount of carbohydrates.

The range of 3.5–5.5 mmol/L is formally considered to be normal for fasting glycemia over almost the entire lifetime of a healthy individual. The exception is the period after birth, when glycemia drops and can range from 1.4 to 6.2 mmol/L. However, within approximately 72 hours it stabilizes within the normal range mentioned above (6.7).

The normal range is maintained under physiological conditions by the complex action of several hormones (insulin, glucagon, cortisol and growth hormone). Its value is individual, can vary over time even within a person, and is influenced by some external causes, including the KD.

Carbohydrates were evolutionary considered to be the primary energy substrates for the brain. It was shown that ketones may also be utilized by the brain and under fasting or starvation becomes the main brain energy source.^18^, ^19^

The pool of different metabolic substrates and their utilization was originally described in 1963 as the Randle cycle—now also known as the glucose‐fatty acid‐ketone body cycle—which explains the reciprocal relationship between the rates of glucose and fatty acid oxidation.^20^

If one substrate in the diet decreases, the oxidation of another increases. Thus, if the availability of carbohydrates decreases, the production and utilization of ketones increase.^21^ It is considered proven that the KD reduces glycemic values compared to a normal diet.^22^, ^23^

In this respect, the differences in mean values of fasting and total glycemia in our cohort are consistent with the assumptions.

It is also of both theoretical and practical interest to investigate how glycemia distribution changes with different fat: nonfat ratio. The standard analysis involves mean glycemia comparisons among the fat: nonfat ratio groups (showing that average glycemia decreases with increasing fat: nonfat ratio). Further analysis then checks whether variability (e.g. standard deviation) changes with the fat: nonfat ratio (in fact, it also decreases with the fat: nonfat ratio). However, this is not the end of the story. Flexible estimates of the whole glycemia distribution for different fat: nonfat ratio groups (e.g. via the kernel density approach) allow us to expand upon the simple comparison of the first two moments (means and SD). As clearly visible in Figure 3, in addition to the location and spread, we can see the shape of the glycemia distribution changes profoundly with fat: nonfat ratio. Specifically, the distribution is less right skewed and with extremely high fat: nonfat ratio, it becomes even left skewed. In practical terms, this amounts to interchanging occasional excursions to higher‐than‐usual glycemia with (much more troublesome) occasional excursions to lower‐than‐usual glycemia. It is not only the decrease in average glycemia, but also a tendency to shoot to low values that might be problematic. The net value of both the mean decrease and the change in the distributional shape is that the probability of glycemia being under conventional hypoglycemia thresholds increases dramatically.

Although the measurement method CGM is burdened with a certain degree of imprecision,^24^ the results show a certain proportion of hypoglycemia, especially at higher fat: nonfat ratio. We consider this finding to be the most important. It may indicate that the pro‐compensatory mechanism maintaining glycemia within the normal range, gluconeogenesis, is unable to respond quickly enough to a low carbohydrate food content and maintain glycemia within the normal range. In this respect, it seems probable that participants would benefit from more gradual changes in the ratio of nutrients in the KD.

If we accept that the measurement of glycemia revealed real hypoglycemia, the absence of signs of hypoglycemia in our subjects might be associated with the syndrome of impaired hypoglycemia awareness which is well‐known in diabetic patients.^25^, ^26^ A number of studies in diabetic patients have shown that previous glycemia (as well as physical activity and sleep time) reduces the response to subsequent hypoglycemia.^27^ As asymptomatic hypoglycemia was previously reported in patients on a KD it is, therefore, possible that this process also occurs in our study or in KD in general. From this point of view the syndrome of impaired hypoglycemia awareness could be considered as a form of human adaptation to a lack of carbohydrates in food.

The importance of the study: this is the first study using CGM to measure the dynamics of glycemic changes at the start of a KD in children with intractable epilepsy.

## 5 LIMITATIONS

The study limitations are mainly the limited accuracy of CGM and the small size of the participants. We asked participants or caregivers to record physical activity but as the records were mostly incomplete, this characteristic is not a part of the analysis. Even so, we believe that our results provide important insights into the dynamics of blood glucose concentrations at the introduction of a KD.

## 6 CONCLUSION

Our results show a consistent trend of decreasing glycemic values with increasing fat: nonfat ratio in KD. Conjointly, the risk of hypoglycemia (<3.5 mmol/L) as well as severe hypoglycemia (<2.5 mmol/L) increased markedly, thus signifying that blood glucose levels should be monitored carefully in epilepsy patients during the first days of KD.

## AUTHOR CONTRIBUTIONS

Klára Brožová: Conceptualization, methodology, investigation, data curation, formal analysis, visualization, writing–original draft, writing–review and editing. Juraj Michalec: Methodology, investigation, data curation, writing–review and editing. Marek Brabec: formal analysis, writing–review and editing. Petra Bořilová: investigation, data curation, writing–review and editing. Pavel Kohout: methodology, data curation, writing–review and editing. Jan Brož: Conceptualization, methodology, writing–original draft, writing–review and editing. All authors reviewed the results and approved the final version of the manuscript.

## FUNDING INFORMATION

The paper was supported by MH CZ – DRO (Thomayer University Hospital – TUH, 00064190).

## CONFLICT OF INTEREST STATEMENT

None of the authors has any conflict of interest to disclose. We confirm that we have read the Journal's position on issues involved in ethical publication and affirm that this report is consistent with those guidelines.

## STANDARD PROTOCOL APPROVALS, REGISTRATIONS, AND PATIENT CONSENTS

Thomayer University Hospital, Prague, Czech Republic Ethics committee approved this study. Written informed consent was obtained from the parents or legal guardians for all patients upon study recruitment.

## Data Availability

The data that support the findings of this study are available from the corresponding author, upon reasonable request.
